# Neuroprotective Effect and Mechanism of Thiazolidinedione on Dopaminergic Neurons* In Vivo* and* In Vitro* in Parkinson's Disease

**DOI:** 10.1155/2017/4089214

**Published:** 2017-03-05

**Authors:** Yanqin Wang, Weilin Zhao, Ge Li, Jinhu Chen, Xin Guan, Xi Chen, Zhenlong Guan

**Affiliations:** ^1^Department of Physiology, College of Life Science, Hebei Normal University, Shijiazhuang, Hebei 050024, China; ^2^Human Movement Science, Hebei Institute of Physical Education, Shijiazhuang, Hebei 050041, China; ^3^Department of Endocrinology, Hebei General Hospital, Shijiazhuang, Hebei 050051, China

## Abstract

The aim of the present study was to gain insight into the neuroprotection effects and mechanism of thiazolidinedione pioglitazone in both* in vitro* and* in vivo* MPP^+^/MPTP induced PD models.* In vivo* experimental results showed that oral treatment of pioglitazone resulted in significant improvements in behavior symptoms damaged by MPTP and increase in the survival of TH positive neurons in the pioglitazone intervention groups. In addition, oral treatment of pioglitazone increased the expression of peroxisome proliferator-activated receptor-*γ* coactivator of 1*α* (PGC-1*α*) and increased the number of mitochondria, along with an observed improvement in mitochondrial ultrastructure. From* in vitro* studies, 2,4-thiazolidinedione resulted in increased levels of molecules regulated function of mitochondria, including PGC-1*α*, nuclear respiratory factor 1 (NRF1), NRF2, and mitochondria fusion 2 (Mfn2), and inhibited mitochondria fission 1 (Fis1). We show that protein levels of Bcl-2 and ERK were reduced in the MPP^+^-treated group compared with the control group. This effect was observed to be reversed upon treatment with 2,4-thiazolidinedione, as Bcl-2 and ERK expression levels were increased. We also observed that levels of the apoptotic protein Bax showed opposite changes compared to Bcl-2 and ERK levels. The results from this study confirm that pioglitazone/2,4-thiazolidinedione is able to activate PGC-1*α* and prevent damage of dopaminergic neurons and restore mitochondria ultrastructure through the regulation of mitochondria function.

## 1. Introduction

Parkinson's disease (PD) is a neurodegenerative disease characterized by progressive degeneration and death of dopaminergic neurons in the substantia nigra compacta (SNc). Dopaminergic neuronal death results in imbalance in nigrostriatal dopamine transmission, leading to severe motor symptoms, including resting tremor, muscle rigidity, bradykinesia, and postural instability [1, 2]. Current treatment options for PD patients include the use of levodopa and nondopaminergic treatments [3, 4]. These drugs alleviate only the symptoms associated with PD and do not prevent the degeneration of dopaminergic neurons. Thus, progression of the disease is not halted, and the therapeutic effect observed with these treatments diminishes over time as the disease progresses. Numerous studies have shown that mitochondrial dysfunction and oxidative stress both play a vital role in the onset and progression of PD [5, 6]. Mitochondria play a key role in maintaining proper cellular physiology, as these organelles are essential for the maintenance of cellular bioenergetics and modulation of the threshold for cell death. Therefore, cellular mechanisms responsible for the regulation of mitochondria quality control are gaining interest. We hypothesize that mitochondria could act as a potential drug target for both prophylaxis and the treatment of PD.

Recently, the nuclear transcription coactivator peroxisome proliferator-activated receptor-*γ* coactivator of 1*α* (PGC-1*α*) has gained increased interest across numerous fields of research. This protein plays an important role in the regulation of mitochondrial biogenesis. Increasing evidence has suggested that expression of PGC-1*α* is reduced in the substantia nigra of PD patients [7]. Additional studies have shown that peroxisome proliferator-activated receptor-gamma agonists demonstrated protective effects in a PD model [8–10]. The protective mechanisms were shown to be attributed to anti-inflammatory [9] and antioxidative [11] effects, but the specific mechanism is not clear. We hypothesize that thiazolidinedione activates expression of PGC-1*α*, increases levels of PGC-1*α*, and provides neuroprotective effects through the regulation of mitochondria function. Peroxisome proliferator-activated receptor-*α*, peroxisome proliferator-activated receptor-*β*, and peroxisome proliferator-activated receptor-*γ* are members of the nuclear receptor family activated by ligands. Thiazolidinediones (TZDs) are drugs which are synthetic high affinity ligands of peroxidase proliferation-activated receptor-*γ* (PPAR-*γ*), including troglitazone, rosiglitazone, and pioglitazone [12]. Currently, troglitazone and rosiglitazone have been shown to present limitations due to safety concerns and have been withdrawn from the market [13, 14]. Therefore, we use pioglitazone in this study, which is a drug used in clinic to treat type 2 diabetes. This drug is a PPAR-*α* and PPAR-*γ* agonist [15], mainly the agonist of PPAR-*γ*. To understand whether the PPAR-*γ* agonist, thiazolidinedione, regulates the expression levels of PGC-1*α*, resulting in improved mitochondria function, we used both* in vitro* and* in vivo* MPP^+^/MPTP PD models. With these models, we tested the expression levels of molecules known to be involved in mitochondria regulation, including PGC-1*α*, nuclear respiratory factor 1 (NRF1), nuclear respiratory factor 2 (NRF2), mitochondrial fission 1 (Fis1), and mitochondrial fusion 2 (Mfn2).

## 2. Experimental Procedure

### 2.1. Animals

Experiments were performed using male mice (C57BL/6) weighing between 23 and 25 g (purchased from the Experimental Animal Center of Hebei Medical University). All animal experiments were carried out in accordance with the National Institute of Health Guide for the Care and Use of Laboratory Animals (NIH Publications number 80-23). Hebei Normal University Ethical Committee has approved the experiments. All efforts were made to minimize animal suffering and reduce the number of animals used in this study.

### 2.2. Chemicals

MPTP, MPP^+^, 2,4-thiazolidinedione (2,4-TZD), and rabbit anti-TH (tyrosine hydroxylase, TH; product number: T8700) polyclonal antibody were all purchased from Sigma-Aldrich. Pioglitazone Hydrochloride Tablets were purchased from Takeda Pharmaceutical Company Limited. Mouse anti-PGC-1*α* monoclonal antibodies (product number: ST1202) were purchased from Millipore. Rabbit anti-NRF1 polyclonal antibody (catalog number: 12482-1-AP), rabbit anti-NRF2 polyclonal antibody (catalog number: 21542-1-AP), rabbit anti-Fis1 polyclonal antibody (catalog number: 10956-1-AP), and rabbit anti-Mfn2 polyclonal antibody (catalog number: 12186-1-AP) were purchased from Proteintech. Rabbit anti-Bcl-2 polyclonal antibody (catalog number: YT5756) and rabbit anti-Bax polyclonal antibody (catalog number: YT0459) were purchased from ImmunoWay Biotechnology Company. Mouse anti-ERK monoclonal antibody (catalog number: AM2189b) was bought from Abgent Wuxi Co. For fluorescent labeling, secondary antibodies conjugated to fluorescein isothiocyanate (FITC) or tetramethyl rhodamine isothiocyanate (TRITC) were used (1 : 300; GSGB-Bio). Rabbit anti-*β*-actin primary antibody was purchased from Bioss Company. Horseradish peroxidase (HRP) conjugated goat anti-rabbit IgG (dilution 1 : 5,000, catalog number: ZB2301) or anti-mouse IgG (1 : 5,000; catalog number: 106912) secondary antibodies were purchased from Beijing ZSGB-Bio Co.

### 2.3. Animal Grouping

The animals were randomly divided into six groups: control group, pioglitazone (Pio) group, MPTP group (MPTP), 10 mg/kg intervention group (MPTP + 10 mg/kg Pio), 20 mg/kg intervention group (MPTP + 20 mg/kg Pio), and 40 mg/kg intervention group (MPTP + 40 mg/kg Pio). The mice in control group received intraperitoneal injection of saline once daily from day 10 to day 14 and intragastric injection of water for 17 days. In Pio group, the other conditions remain unchanged except that water is replaced with 40 mg/kg Pio. The MPTP-treated mice received intraperitoneal injection of MPTP dissolved in saline (30 mg/kg body weight) once daily for 5 days (from day 10 to day 14), and water was administrated intragastrically for 17 days. Mice in the three intervention groups were given intragastrically appropriate doses of Pio for 17 d according to grouping, and from day 10 to day 14, mice were given intraperitoneally the same doses of MPTP as those in MPTP group for 5 days. Pioglitazone was dissolved in drinking water for the pioglitazone intervention groups and pioglitazone group.

Behavioral tests (free-standing rear, rotarod, and swim) were performed before the first and after the second hours following the final MPTP injection. Animals were sacrificed three days following the final MPTP injection. Immunofluorescence studies were carried out to detect protein localization and for the semiquantitative analysis of changes in TH and PGC-1*α* levels. Six animals were used per group. For protein expression and mitochondria ultrastructure studies, four animals were used per group.

### 2.4. Cell Culture Treatment

SH-SY5Y cells were cultured in Dulbecco's modified Eagle medium containing 10% fetal bovine serum (FBS). The cultures contained 1% penicillin/streptomycin and were maintained in a humidified atmosphere containing 5% CO_2_ at 37°C. Cells were plated at 1 × 10^5^ cells/mL in 10 cm culture dishes overnight. They were then randomly assigned into the control group, MPP^+^ group, and the 2,4-thiazolidinedione(2,4-TZD) intervention groups (0.01, 0.1, 1, and 10 *μ*M). For the control group, DMEM basic medium was added. A concentration of 1 mmol/L MPP^+^ (IC_50_) was used in the MPP^+^-treatment and 2,4-TZD intervention groups. MPP^+^ and different doses of 2,4-TZD were added to the cells at the same time. Cells were incubated with treatments for 24 hours. Following the treatment period, proteins were extracted for Western blot experiments.

### 2.5. Behavior Assessment

#### 2.5.1. Locomotor Activity

To observe mice's behavioral changes in the familiar and closed, quiet, and dark environment, mice were placed in the central part of autonomous activity tester and the free-standing number was recorded within 5 minutes.

#### 2.5.2. Swim Test

In the swim test, mice were placed in water tubs according to the methods described in Donnan et al. [16] and Haobam et al. [17], where the water depth was 40 cm and the temperature was maintained between 35°C and 36°C. Following the 2-minute test, mice were blow-dried and were returned to their cages. The retention time of mice floating with their head on the water surface with no sliding of limbs was recorded within 2 minutes.

#### 2.5.3. Rotarod Test

In order to study the movement balance ability and the limb muscle strength of mice, we carried out the rotarod test. Mice were made to walk on a rotating rod with a rod speed of 30 rpm. The retention time on the rod was recorded within 5 minutes.

### 2.6. Immunofluorescence

On the third day of the last intraperitoneal MPTP injection, animals were deeply anesthetized with injection of sodium pentobarbital (40 mg/kg, i.p.) and perfused transcardially using a volume of 10 mL of saline, followed by a volume of 40 mL of phosphate buffer (PB; pH 7.4) containing 4% paraformaldehyde. Brains were removed immediately and placed in 0.1 M PB containing 20% sucrose overnight at 4°C. The next day, midbrain samples containing SN were serially cut into coronal sections of 30 *μ*m thickness using a freezing microtome. Two sets of sections were incubated with rabbit anti-TH IgG (1 : 5,000 dilution) and mouse anti-PGC-1*α* IgG (1 : 1,000 dilution) for 24 hours at room temperature in 0.01 M PBS containing 2% normal donkey serum and 0.1% Triton X-100. The sections were then rinsed in 0.01 M PBS and then incubated with TRITC-conjugated goat anti-mouse IgG or goat anti-rabbit IgG (1 : 300 dilution) in antibody diluent for 4 hours at room temperature. Following a wash step with PBS, the sections were mounted on gelatin-coated glass slides. Slides were covered with 0.01 M PBS containing 50% glycerine and examined under a fluorescence microscope (Olympus BX51).

### 2.7. Transmission Electron Microscope Analysis

On the third day of the last intraperitoneal MPTP injection, animals were deeply anesthetized with injection of sodium pentobarbital (40 mg/kg, i.p.) and perfused transcardially with a volume of 10 mL of saline, followed by a volume of 40 mL of phosphate buffer (PB; pH 7.4) containing 4% paraformaldehyde and 2.5% glutaraldehyde. Brains including midbrain SN were removed immediately and placed in paraformaldehyde containing 4% glutaraldehyde and perfused for 24 hours. The tissue samples were then washed three times with 0.1 M phosphate buffer and postfixed in 1% osmium tetroxide for 1.5 hours, followed by dehydration using a graded alcohol series. Samples were then embedded in epoxy resin and allowed to solidify for 26 hours at 60°C. The region of interest was selected using a stereomicroscope and transmitted light to image the brain sections. Areas of interest were cut into trapezoidal sections using a razor blade for further trimming and sectioning. Tissue samples were then cut into 50 nm ultrathin sections. Sections were then double-stained with 0.5% uranyl acetate and 3% lead citrate and then imaged at 80 kV using a transmission electron microscope (H-7650).

### 2.8. Western Blotting

Western blot was performed to confirm the protein levels of PGC-1*α*, NRF1, NRF2, Fis1, and Mfn2. In addition, Western blot was also used to monitor changes in cell signaling using markers such as Bcl-2, Bax, and ERK. In brief, SH-SY5Y cells were collected and put in an Eppendorf tube, and then cells were centrifuged for 15 min at 4°C, and the supernatant was removed. Cold Radio-Immunoprecipitation Assay (RIPA) lysis buffer (1 mL RIPA containing 10 *μ*L phenylmethanesulfonyl fluoride) was then added to lyse the cells. The cell homogenate was centrifuged at 12,000 rpm for 20 min at 4°C to remove cell debris. The protein concentration was measured using an ultra-micro ultraviolet-visible spectrophotometer. Gel samples containing 50 *μ*g of total cellular protein were boiled for 10 min and then separated on a 10% SDS-polyacrylamide gel by gel electrophoresis. Samples within the gel were transferred onto PVDF (polyvinylidene fluoride) membrane (Millipore). The membranes were then incubated with blocking buffer containing 5% nonfat milk for 2 hours to block nonspecific binding sites. Membranes were then incubated with rabbit anti-TH (1 : 5,000 dilution), mouse anti-PGC-1*α* (1 : 1,000 dilution), rabbit anti-NRF1 (1 : 500 dilution), rabbit anti-NRF2 (1 : 1,000 dilution), rabbit anti-Fis1 (1 : 500 dilution), rabbit anti-Mfn2 (1 : 1,000 dilution), rabbit anti-Bcl-2 (1 : 1,000 dilution), rabbit anti-Bax (1 : 1,000 dilution), or mouse anti-ERK IgG (1 : 1,000 dilution) at 4°C overnight. Membranes were washed three times with Tris-Buffered Saline and Tween 20 (TBST) for 30 minutes. Then membranes were incubated with their corresponding secondary antibodies (HRP conjugated anti-mouse or anti-rabbit IgG) for 2 hours at room temperature. Membranes were washed three times with TBST for 30 minutes again. Protein bands were visualized via an enhanced chemiluminescence method using an ECL kit. Bands were scanned using a scanner and quantified using Quantity One software.

### 2.9. Data Collection and Statistical Analysis

For semiquantitative analysis, TH-positive neurons in SNc and PGC-1*α*-positive cells in substantia nigra (SN) were counted in each slide. There were five slides of each mouse, and each group had six mice. Image-Pro Plus 6.0 was used to count TH- and PGC-1*α*-positive cells. The numbers of positive cells were expressed as mean ± SD. Statistical analysis was performed with the ANOVA followed by LSD test for parametric analysis. Statistical analysis was performed using SPSS version 13.0, with the alpha level of significance set to 0.05.

## 3. Results

### 3.1. Behavior Assessment

**Figure 1:**
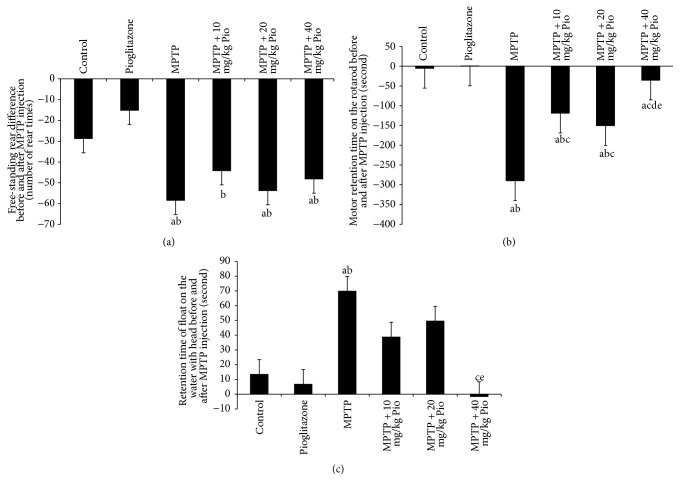
Effect of pioglitazone on behavior in MPTP-treated C57BL/6 mice. “a” indicates *P* < 0.05, compared with control group; “b” indicates *P* < 0.05, compared with pioglitazone group; “c” indicates *P* < 0.05, compared with MPTP group; “e” indicates *P* < 0.05, compared with MPTP + 20 mg/kg Pio group.

In order to study the effect of pioglitazone on motor and balance ability in MPTP-treated mice, behavioral studies were carried out. We used the free-standing rear, exercise on a rotating rod, and swimming after continuous administration of MPTP (once per day) behavioral tests. We show that the MPTP-treated mice exhibited significant reduction in rearing frequency compared to control mice (Figure 1(a)). The exercise retention time on a rotating rod was also found to be significantly reduced in MPTP-treated mice (Figure 1(b)). However, the float time on the water surface with no sliding of limbs was observed to increase compared to control mice (Figure 1(c)). Rearing frequency, swimming time, and running time on the rotarod were all found to be increased in intervention groups. Animals in pioglitazone group exhibited performance consistent with control animals.

### 3.2. Pioglitazone Increased the Survival of TH-Positive Neurons in SNc Damaged by MPTP

**Figure 2:**
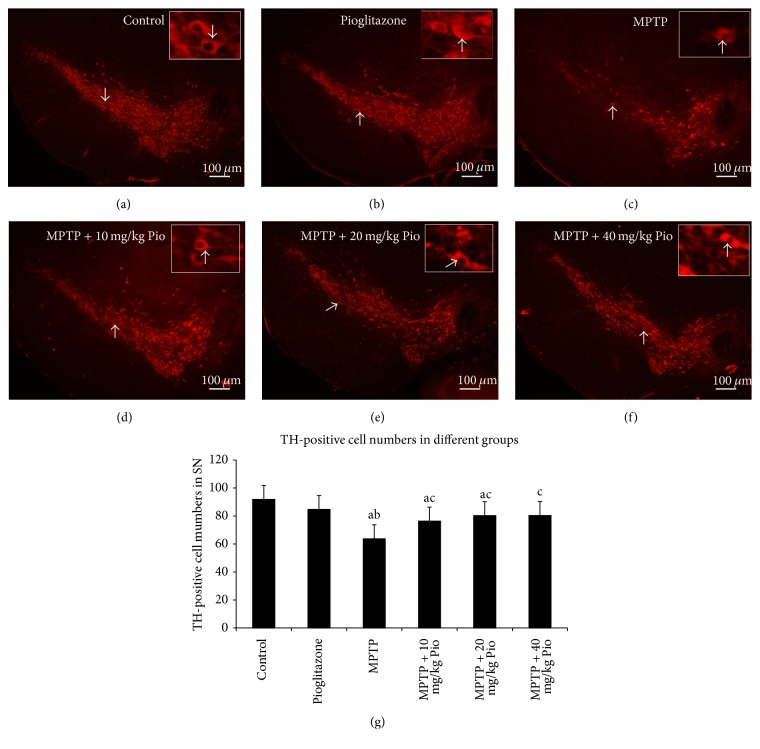
TH-positive neurons in different groups. “a” indicates *P* < 0.05, compared with control group; “b” indicates *P* < 0.05, compared with pioglitazone group; “c” indicates *P* < 0.05, compared with MPTP group. The bars in (a)–(f) indicate 100 *μ*m; the arrows in each picture show the same neurons.

The integrity of the nigrostriatal system was assessed through analysis of the number of dopaminergic neurons in the SNc by TH immunofluorescence. The number of TH-positive neurons was found to be dramatically decreased in mice that received continuous administration of MPTP compared to both control and pioglitazone groups (Figure 2(g)). Oral administration of pioglitazone prior to MPTP treatment was found to result in an increase in the number of TH-positive neurons compared to MPTP mice (Figures 2(d)–2(f)). However, no statistical difference was detected between the three intervention groups (Figure 2(g)). Additionally, no notable changes in the number of TH-positive neurons were observed between the control mice and mice in pioglitazone group.

### 3.3. Pioglitazone Increased the PGC-1*α* Expression

**Figure 3:**
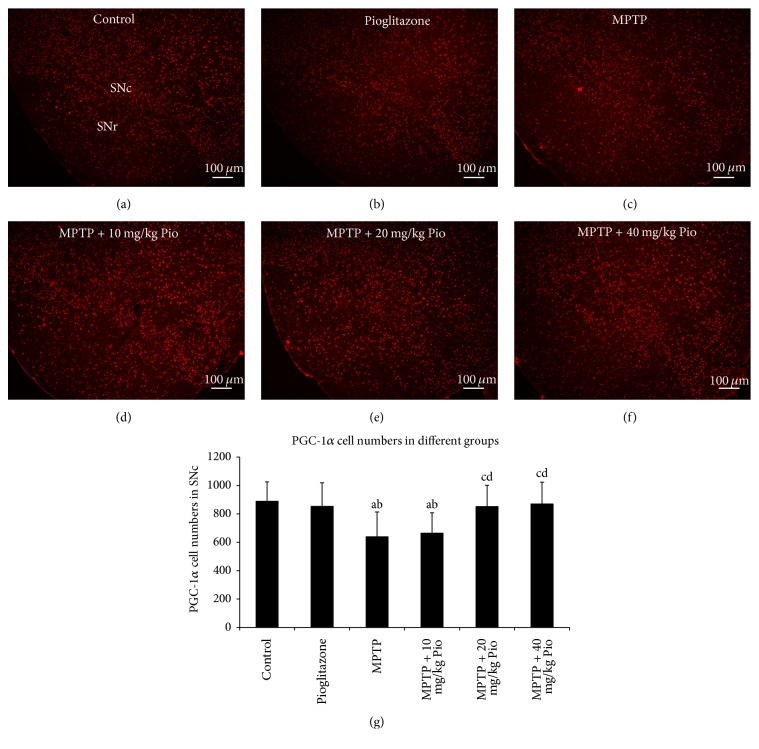
Effect of pioglitazone on PGC-1*α* expression in MPTP C57BL/6 mice. “a” indicates *P* < 0.05, compared with control group; “b” indicates *P* < 0.05, compared with pioglitazone group; “c” indicates *P* < 0.05, compared with MPTP group; “d” indicates *P* < 0.05, compared with MPTP + 10 mg/kg Pio group. The bars in (a)–(f) indicate 100 *μ*m.

PGC-1*α* is the primary transcription factor involved in the regulation of mitochondrial biogenesis. We used immunofluorescence to gain insight into neuronal mitochondrial function in the SN. Specifically, we analyzed changes in the levels of PGC-1*α* to understand the cellular target of pioglitazone-mediated neuroprotection in our PD model. We did not detect a difference in PGC-1*α* expression levels between the control group and the pioglitazone group. However, the number of PGC-1*α*-positive cells was observed to be reduced following MPTP treatment, an effect that could be reversed with pioglitazone treatment. However, we did not observe a statistical difference between MPTP group and the 10 mg/kg pioglitazone intervention group, and there is no difference between 20 mg/kg pioglitazone intervention group and the 40 mg/kg pioglitazone intervention group (Figure 3) (*P* > 0.05).

### 3.4. The Effect of Pioglitazone on Mitochondrial Ultrastructure

**Figure 4:**
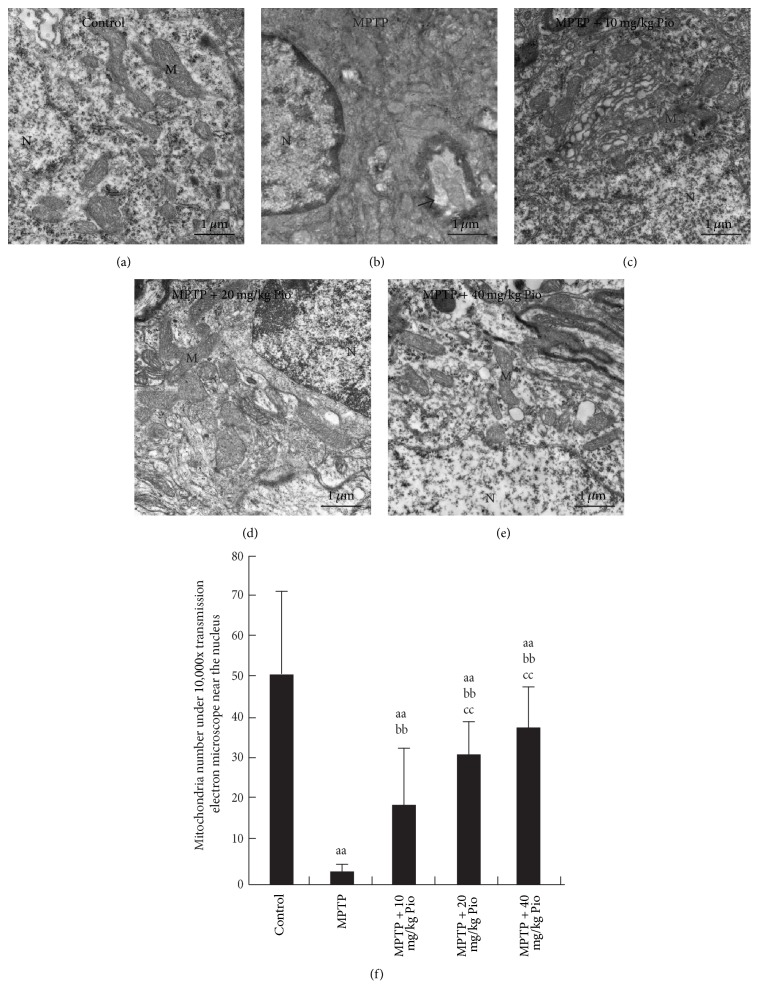
Effect of pioglitazone on the mitochondrial ultrastructure in SNc of MPTP C57BL/6 mice. “aa” indicates *P* < 0.01, compared with control group; “bb” indicates *P* < 0.01, compared with MPP^+^ group; “cc” indicates *P* < 0.01, compared with 0.01 *μ*mol/L 2,4-TZD group. N indicates nucleus; M indicates mitochondria. Arrow points to the demyelination in (b). The bars in (a)–(e) indicate 1 *μ*m.

Transmission electron microscopy (TEM) was utilized to observe changes in mitochondrial ultrastructure in SNc of each treatment group.  Figure 4 shows the presence of many mitochondria surrounding the nucleus in samples from the control group, with no swelling phenomenon observed. Additionally, the mitochondria membranes were found to be integrated, and mitochondrial cristae were observed. However, in MPTP group, virtually no mitochondria were observed surrounding the nucleus, and the nuclei were seen to be shrinking with a severe demyelination phenomenon. The mitochondria number increased (Figure 4(f)) and mitochondria ultrastructure was observed to improve following pioglitazone treatment (Figures 4(c)–4(e)).

### 3.5. The Effect of 2,4-Thiazolidinedione (2,4-TZD) on PGC-1*α*, NRF1, and NRF2 Protein Levels

**Figure 5:**
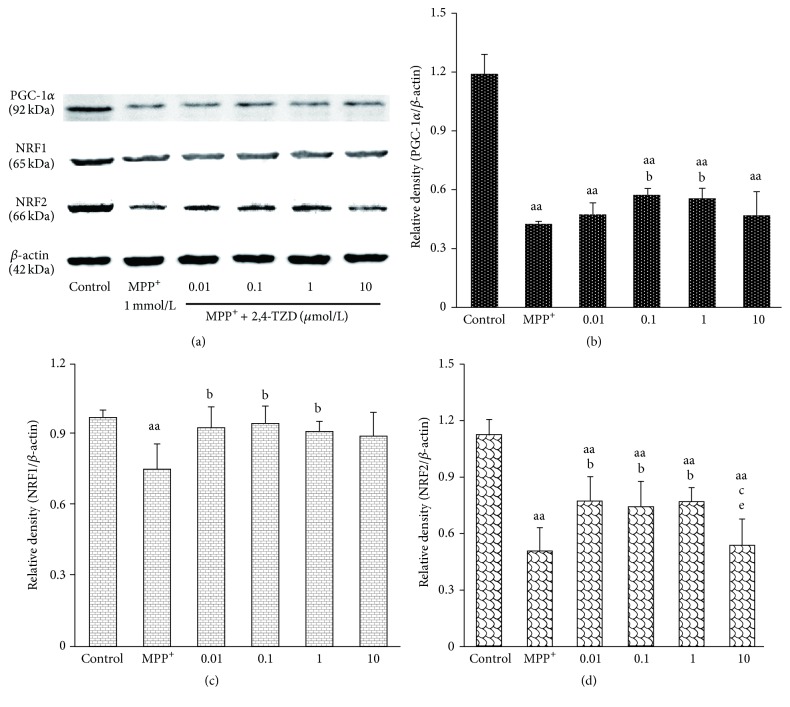
Effect of thiazolidinedione on PGC-1*α*, NRF1, and NRF2 protein levels. (a) The expression of PGC-1*α*, NRF1, and NRF2 in different groups; *β*-actin was used as the loading control. ((b)–(d)) PGC-1*α*, NRF1, and NRF2 protein levels. “aa” indicates *P* < 0.01, compared with control group; “b” indicates *P* < 0.05, compared with MPP^+^ group; “c” indicates *P* < 0.05, compared with 0.01 *μ*mol/L 2,4-TZD group; “e” indicates *P* < 0.05, compared with 1 *μ*mol/L 2,4-TZD group.

Due to pioglitazone is the solid dosage forms, it is difficult to dissolved in the culture medium, so we used the powdered form of its drug intermediates 2,4-thiazolidinedione (2,4-TZD) to examine the mechanism of pioglitazone protection in SN dopaminergic neurons. Through Western blot, we revealed that PGC-1*α* protein levels were reduced in the MPP^+^ group compared to the control group (*P* < 0.01). In addition, we found that intervention with 2,4-TZD treatment resulted in increased PGC-1*α* protein levels in the case of 0.1 and 1 *μ*mol/L 2,4-TZD treatment groups (Figures 5(a) and 5(b)). However, these increased levels were still lower than the control group.

We next focused on understanding if 2,4-TZD plays protection role through downstream signals of PGC-1*α*, such as NRF1 and NRF2. We show that NRF1 and NRF2 protein levels were decreased in the MPP^+^ group compared to the control group (*P* < 0.01). However, these levels were increased in the 0.01, 0.1, and 1 *μ*mol/L 2,4-TZD treatment groups compared to the MPP^+^ group (*P* < 0.05). No significant differences were observed between the three 2,4-TZD intervention groups. In addition, 10 *μ*mol/L 2,4-TZD was not observed to have a discernible effect on protein levels (Figures 5(a), 5(c), and 5(d)).

### 3.6. The Effect of 2,4-TZD on Mitochondria Fission and Fusion

**Figure 6:**
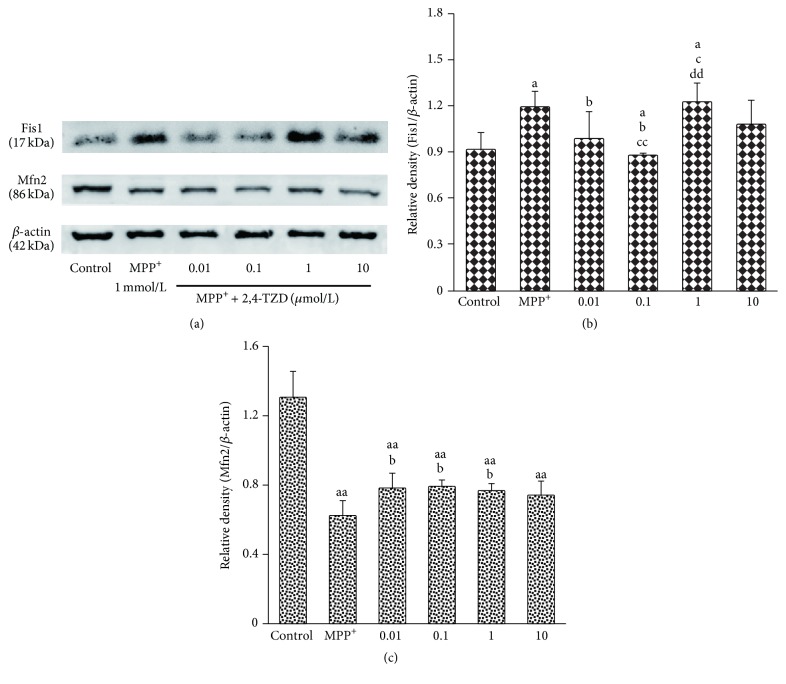
Effect of thiazolidinedione on mitochondria fission and fusion. (a) The expression of Fis1 and Mfn2 in different groups; *β*-actin was used as the loading control. (b) and (c) express the protein level of Fis1 and Mfn2. “a” indicates *P* < 0.05 and “aa” indicates *P* < 0.01, compared with control group; “b” indicates *P* < 0.05, compared with MPP^+^ group; “c” indicates *P* < 0.05 and “cc” indicates *P* < 0.01, compared with 0.01 *μ*mol/L 2,4-TZD group; “dd” indicates *P* < 0.05, compared with 0.1 *μ*mol/L 2,4-TZD group.

To evaluate the effect of 2,4-TZD on mitochondria function, we analyzed mitochondria function related protein fission (Fis1) and fusion (Mfn2) expression levels by immunoblotting. Fis1 levels in the MPP^+^ group were significantly higher compared to those in the control group (*P* < 0.05). In addition, 2,4-TZD intervention was observed to reduce Fis1 protein levels, with a significant difference observed between the 0.01 and 0.1 *μ*mol/L 2,4-TZD-treated groups compared to that of the MPP^+^ group (*P* < 0.05). No statistical difference was observed between the 1 *μ*mol/L 2,4-TZD-treated group and the MPP^+^ group (Figures 6(a) and 6(b)).

Mfn2 protein levels were observed to decline following MPP^+^ treatment compared to the control group (Figures 6(a) and 6(c)). Upon treatment with 0.01, 0.1, or 1 *μ*mol/L 2,4-TZD, the Mfn2 protein levels were found to significantly increase compared to the MPP^+^ group (*P* < 0.01), with no difference observed between the three 2,4-TZD intervention groups.

### 3.7. The Effect of 2,4-TZD on the Expression of Bax, Bcl-2, and ERK

**Figure 7:**
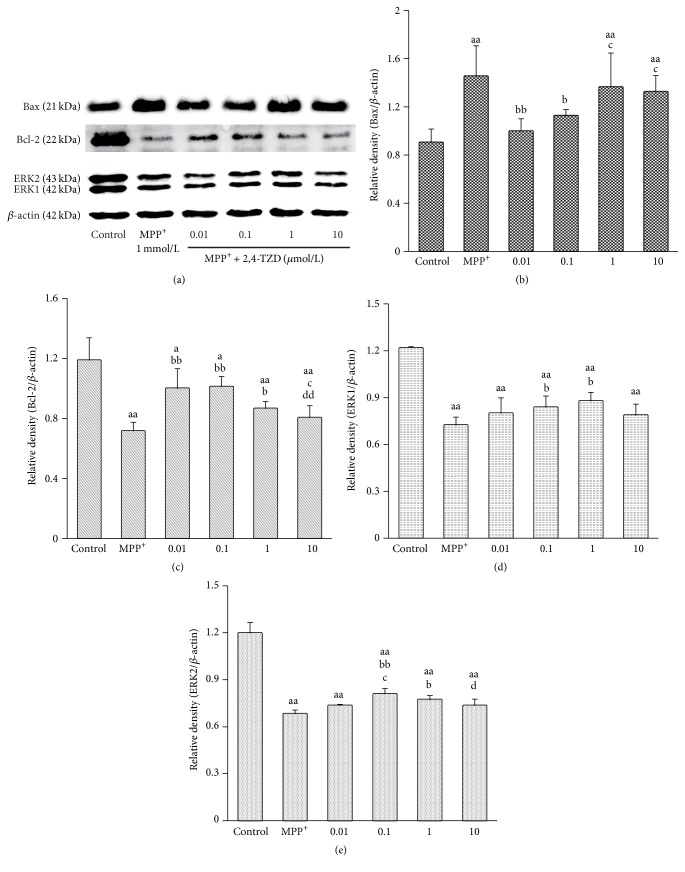
Effect of thiazolidinedione on apoptosis and levels of antiapoptosis proteins. (a) The expression of Bax, Bcl-2, and ERK in different groups; *β*-actin was used as the loading control. (b)–(e) express the protein levels of Bax, Bcl-2, and ERK. “a” indicates *P* < 0.05 and “aa” indicates *P* < 0.01, compared with control group; “b” indicates *P* < 0.05 and “bb” indicates *P* < 0.01, compared with MPP^+^ group; “c” indicates *P* < 0.05 and “cc” indicates *P* < 0.01, compared with 0.01 *μ*mol/L 2,4-TZD group; “d” indicates *P* < 0.05 and “dd” indicates *P* < 0.01, compared with 0.1 *μ*mol/L 2,4-TZD group.

In order to monitor the signaling pathway changes, Bax, Bcl-2, and ERK protein levels were detected by Western blot. Bax levels were found to be increased in the MPP^+^ group compared to the control group. However, Bax protein levels were observed to be reduced following lower concentration 2,4-TZD treatment. In contrast to the changes observed in Bax levels, protein levels of Bcl-2 and ERK displayed the opposite changes (Figure 7). The results presented here indicate that 0.01 and 0.1 *μ*mol/L 2,4-TZD treatment exhibited enhanced protective effects.

## 4. Discussion

Pioglitazone is a thiazolidinedione medicine, which has gained increasing interest due to its anti-inflammatory [9, 18] and antioxidant properties [19] in neurodegenerative disorders, such as PD [20–22]. Inflammation and oxidative stress are closely related to mitochondria function, and PGC-1*α* is the key factor involved in the regulation of mitochondrial biogenesis. So we want to know if pioglitazone could regulate mitochondria function damaged by MPTP and MPP^+^ in dopaminergic neurons.

In our study, we observed that MPTP-treated mice exhibited abnormal motor abilities, such as a reduced rearing frequency (indicating decreased function of the central nervous system), a reduced exercise retention time on the rotating rod (indicating deficits in motor balance and muscle coordination), and a prolonged float time on the water surface with no sliding of limbs (indicative of motor dysfunction). These observations are similar to those reported in previous studies [23, 24]. However, upon administration of pioglitazone for 17 days, we observed that the motor abilities of mice mentioned above were improved compared to MPTP-treated mice. We observed similar results with other studies carried out with pioglitazone, where the anti-inflammatory properties associated with pioglitazone pretreatment were observed to reduce expression levels of glial fiber acid protein (GFAP) (data not shown) [20], increase the number of TH-positive neurons in the SNc, and rescue deficits in motor performance. In our current study, we found that pretreatment with pioglitazone resulted in changing of mitochondria ultrastructure in dopaminergic cells in the SNc following MPTP damage. Inflammation and ROS both affect mitochondria function, which is the critical node for cell survival and apoptosis. Therefore, the target of protective effects of pioglitazone may be involved in regulating mitochondrial function through PGC-1*α*.

Mitochondria are responsible for supplying energy to cells. PGC-1*α* is the primary regulatory factor of mitochondria biogenesis, as it is able to regulate mitochondria replication and transcription factors, such as NRF1 and NRF2 [1]. These factors can combine with mitochondrial gene promoters, such as *β*-ATP synthase, cytochrome C oxidation subunits IV, and mitochondrial transcription factor A (mtTFA). mtDNA is important for the synthesis of enzymes involved in the mitochondrial respiratory chain. There was a research that described the importance of PGC-1*α* in both the maintenance and repair of mitochondrial energy metabolism [7]. This study also showed that PGC-1*α* expression levels were lower in substantia nigra dopaminergic neurons of PD patients. However, activation of PGC-1*α* expression resulted in increased expression levels of mitochondrial respiratory chain encoding subunits, rescuing the loss of DA neurons induced by alpha-synuclein and rotenone. In addition, another study showed that knock-down of the PGC-1*α* gene augmented the toxic injury associated with alpha-synuclein aggregation [25]. Here, we show that PGC-1*α* expression was reduced following MPTP/MPP^+^ treatment, an effect seen to be rescued with an intervening treatment of pioglitazone or 2,4-thiazolidinedione. Additionally, we observed changes in NRF1 and NRF2 levels which correlated with changes in PGC-1*α* levels. Some researchers showed that the zinc finger protein, PARIS (ZNF746), was found to accumulate in the human PD brain [1, 26]. This was found to result in reduced expression levels of PGC-1*α*, which subsequently inhibited NRF1, a protein important in mitochondrial function and oxidant metabolism. Existing data suggest that PGC-1*α* overexpression leads to increased mitochondrial biogenesis in a myocardial cell model [26, 27]. In addition, PGC-1*α* knockout mice have been shown to have a reduced number of mitochondria, as well as a reduced mitochondrial respiratory capacity in slow-twitch muscle fibers [26].

Mitochondria comprise a network that constantly undergoes fission and fusion to maintain balance. We studied mitochondrial fission and mitochondrial fusion to assess function of mitochondria. From our studies, we showed that a certain concentration range of 2,4-thiazolidinedione prevented cell death (data shown in Supplementary Material available online at https://doi.org/10.1155/2017/4089214). We observed significantly reduced ratio of Fis1/Mfn2 in MPP^+^ group, suggesting that the balance between fission and fusion was broken. However a certain concentration range of 2,4-thiazolidinedione resulted in an improvement between mitochondrial fission and fusion in cells damaged by MPP^+^. Mitochondrial fission and fusion play critical roles in the maintenance of quality of mitochondria. Mitochondrial fusion reduces stress by mixing the contents of partially damaged mitochondria as a form of complementation. Fission is a critical process required for the creation of new mitochondria, while also contributing to quality control mechanisms by promoting the removal of damaged mitochondria. Previous studies have shown that overexpression of Fis1 resulted in increased mitochondrial fragmentation, which ultimately led to apoptosis or triggered autophagy [28, 29]. There is one study which shows that expression of Mfn2 is driven by PGC-1*α*/ERR*α*, both of which activate Mfn2 transcription activity [30]. Researchers found that Mfn2 activation promotes the mitochondrial fusion and functions to protect mitochondria from free radical damage [31]. These studies had similar results to what was observed in our study.

Previous studies have demonstrated that rosiglitazone exhibits protective effects in SH-SY5Y cells, protecting cells from apoptosis by inducing the expression of antioxidant enzymes and regulating Bcl-2 and Bax expression [11, 32]. Results from our study showed that expression of Bax, a protein involved in mitochondria apoptosis signaling, was increased in MPP^+^ group. We also observed that expression levels of the antiapoptotic protein, Bcl-2, were reduced. However, treatment with 2,4-thiazolidinedione was observed to reverse these effects, reducing Bax and increasing Bcl-2 protein levels significantly. A similar trend was observed with ERK as Bcl-2, indicating the cell survival state. The results suggest that the neuroprotective role of 2,4-TZD acts through the regulation of function of mitochondria. PGC-1*α* is activated by 2,4-TZD and may play roles through its antioxidative properties. Previous study found that PGC-1*α* was an essential factor for expression of several important antioxidant enzymes, including GPx1, Cu/ZnSOD, and MnSOD, which are responsible for clearing reactive oxygen species (ROS) [33]. Moreover, whether PGC-1*α* can regulate mitophagy is unclear and needs further research.

## 5. Conclusion

In conclusion, our research suggests that pioglitazone plays a neuroprotective role in dopaminergic neurons in both* in vitro* and* in vivo* PD models. The neuroprotective effect observed was associated with PGC-1*α* activation and the regulation of the expression of proteins involved in mitochondria function, such as NRF1, NRF2, Fis1, and Mfn2.

## Supplementary Material


**Effect of** **2,4-** **thiazolidinedione on the survival rate induced by with MTS assay in SH-SY5Y.** The survival rate was found to be dramatically decreased in MPP^+^ group, 2,4-TZD intervention made the survival rate significantly increased in 0.01, 0.1, 1, and 10 μmol/L 2, 4-TZD treatment groups. The intervention effects were better in 0.01, 0.1, 1μmol/L 2, 4-TZD treatment groups than in 10 μmol/L 2, 4-TZD treatment groups. “aa” indicates *P*< 0.01, compared with control group; “b” indicates *P*< 0.05, compared with MPP^+^group; “bb” indicates *P* < 0.01, compared with MPP^+^ group.
**Comparison of cell apoptosis percentage of each group detected with Hoechst33342 staining.** The apoptosis rate was dramatically increased in group compared to control group, intervention made the apoptosis rate reduced compared to MPP^+^ group. There is no significant difference in 0.01, 0.1, 1 μmol/L 2, 4-TZD treatment group. The apoptosis rate of 10 μmol/L 2, 4-TZD treatment group raised obviously. “aa”indicates *P*< 0.01, compared with control group; “bb” indicates *P*< 0.01, compared with MPP^+^group; “cc” indicates *P* < 0.01, compared with 0.01μmol/L 2, 4-TZD treatment group; “dd” indicates *P* < 0.01, compared with 0.1μmol/L 2, 4-TZD treatment group; “ee” indicates *P* < 0.01, compared with 1μmol/L 2, 4-TZD treatment group.
**Effect of 2, 4-TZD on MPP + induced apoptosis by Fluoro-Jade C(FJC) staining in SH-SY5Y.** FJC is a green fluorescent anion derivatives labeling the degenerating neurons. Under inverted fluorescence microscope, took photos of all cells in the bright field and then took photos of apoptotic cells with FITC channel, finally calculated cell apoptosis rate. The apoptosis rate was dramatically increased in group compared to control group, intervention made the apoptosis rate reduced compared to MPP^+^ group. “aa” indicates *P*< 0.01, compared with control group; “bb” indicates *P*< 0.01, compared with MPP^+^group; “c” indicates *P* < 0.05, compared with 0.01μmol/L 2, 4-TZD treatment group, “cc” indicates *P* < 0.01, compared with 0.01μmol/L 2, 4-TZD treatment group; “dd” indicates *P* < 0.01, compared with 0.1μmol/L 2, 4-TZD treatment group; “ee” indicates *P* < 0.01, compared with 1μmol/L 2, 4-TZD treatment group.
