# Supplementary material for: Neuroprotective Effect and Mechanism of Thiazolidinedione on Dopaminergic Neurons In Vivo and In Vitro in Parkinson's Disease
Source: PPAR Res. 2017 Mar 5;2017:4089214. doi: 10.1155/2017/4089214 (PMC5357540; doi:10.1155/2017/4089214)
Supplement: Supplementary file 1 — Effect of 2,4- thiazolidinedione on the survival rate induced by with MTS assay in SH-SY5Y. The survival rate was found to be dramatically decreased in MPP+ group, 2,4-TZD intervention made the survival rate significantly increased in 0.01, 0.1, 1, and 10 μmol/L 2, 4-TZD treatment groups. The intervention effects were better in 0.01, 0.1, 1μmol/L 2, 4-TZD treatment groups than in 10 μmol/L 2, 4-TZD treatment groups. “aa” indicates P< 0.01, compared with control group; “b” indicates P< 0.05, compared with MPP+group; “bb” indicates P < 0.01, compared with MPP+ group. Comparison of cell apoptosis percentage of each group detected with Hoechst33342 staining. The apoptosis rate was dramatically increased in group compared to control group, intervention made the apoptosis rate reduced compared to MPP+ group. There is no significant difference in 0.01, 0.1, 1 μmol/L 2, 4-TZD treatment group. The apoptosis rate of 10 μmol/L 2, 4-TZD treatment group raised obviously. “aa”indicates P< 0.01, compared with control group; “bb” indicates P< 0.01, compared with MPP+group; “cc” indicates P < 0.01, compared with 0.01μmol/L 2, 4-TZD treatment group; “dd” indicates P < 0.01, compared with 0.1μmol/L 2, 4-TZD treatment group; “ee” indicates P < 0.01, compared with 1μmol/L 2, 4-TZD treatment group. Effect of 2, 4-TZD on MPP + induced apoptosis by Fluoro-Jade C(FJC) staining in SH-SY5Y. FJC is a green fluorescent anion derivatives labeling the degenerating neurons. Under inverted fluorescence microscope, took photos of all cells in the bright field and then took photos of apoptotic cells with FITC channel, finally calculated cell apoptosis rate. The apoptosis rate was dramatically increased in group compared to control group, intervention made the apoptosis rate reduced compared to MPP+ group. “aa” indicates P< 0.01, compared with control group; “bb” indicates P< 0.01, compared with MPP+group; “c” indicates P < 0.05, compared with 0.01μmol/L 2, 4-TZD treatment group, “cc” indicates P < 0. [file 4089214.f1.docx]

**Supplemental Figures and legends**


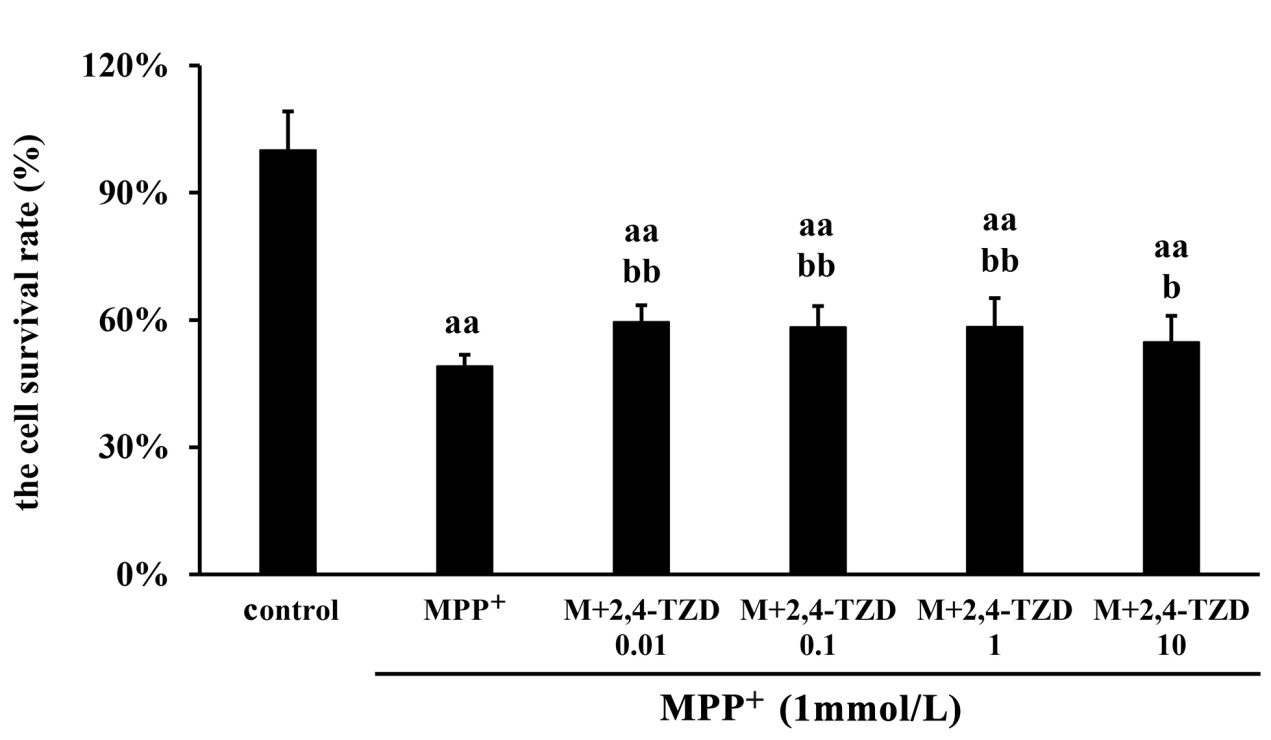


Fig 1 Effect of 2, 4-TZD on the cell viability induced by MPP^+^ by MTS assay

aa indicates *P*< 0.01, compared with control group; b indicates *P*< 0.05 and bb indicates *P*< 0.01, compared with MPP^+^ group.


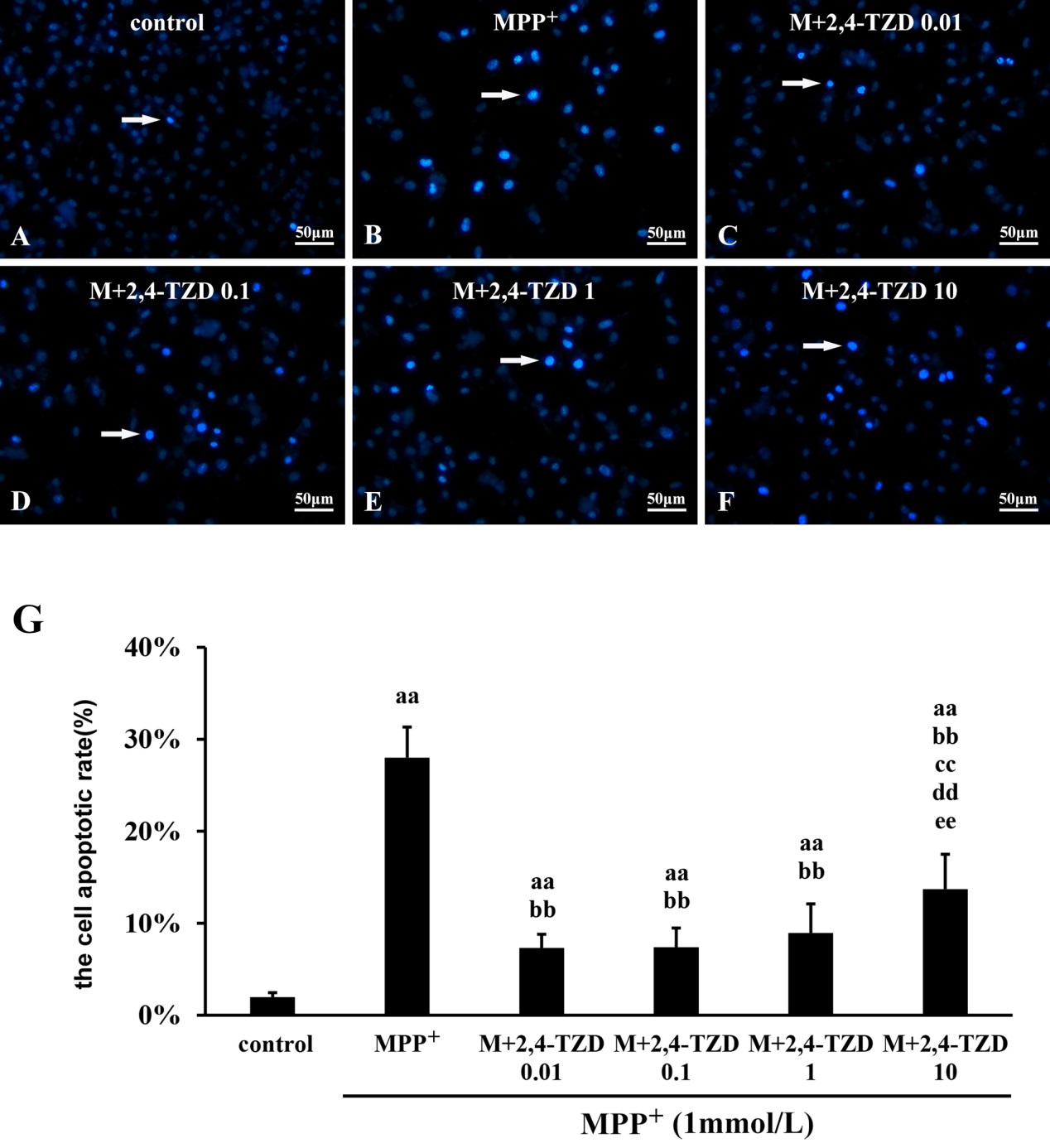


Fig. 2 Effect of 2, 4-TZD on MPP^+^ induced apoptosis by Hoechst33342 staining in SH-SY5Y

aa indicates *P*< 0.01, compared with control group; bb indicates *P*< 0.01, compared with MPP^+^ group; cc indicates *P*<0.01, compared with 0.01 μmol/L 2,4-TZD group; dd indicates *P*< 0.01, compared with 0.1 μmol/L 2,4-TZD group; ee indicates *P*<0.01, compared with 1 μmol/L 2,4-TZD group.


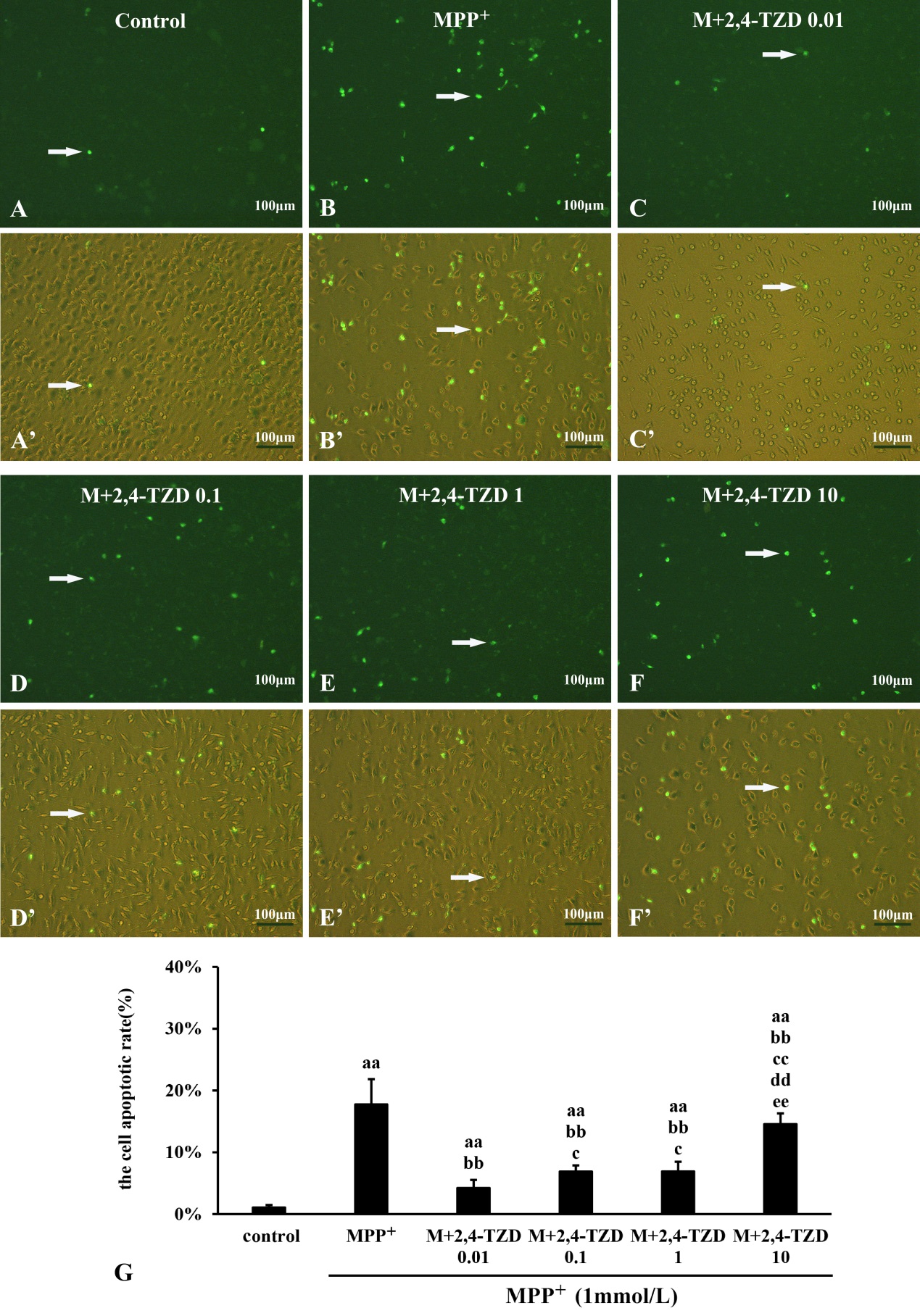


Fig. 3 Effect of 2, 4-TZD on MPP^+^ induced apoptosis by Fluoro-Jade C staining in SH-SY5Y

aa indicates *P*< 0.01, compared with control group; bb indicates *P*< 0.01, compared with MPP^+^ group; c indicates *P*<0.05, compared with 0.01 μmol/L 2,4-TZD group; dd indicates *P*< 0.01, compared with 0.1 μmol/L 2,4-TZD group; ee indicates *P*<0.01, compared with 1 μmol/L 2,4-TZD group. A, B, C, D, E, F are the pictures under the fluorescence, A’, B’, C’, D’, E’, F’ are the corresponding pictures under the bright.
